# Can prior exposure to stress enhance resilience to ocean warming in two oyster species?

**DOI:** 10.1371/journal.pone.0228527

**Published:** 2020-04-10

**Authors:** Roberta R. C. Pereira, Elliot Scanes, Mitchell Gibbs, Maria Byrne, Pauline M. Ross

**Affiliations:** 1 School of Life and Environmental Science, The University of Sydney, Camperdown, NSW, Australia; 2 School of Medical Sciences, The University of Sydney, Camperdown, NSW, Australia; University of Hong Kong, HONG KONG

## Abstract

Securing economically and ecologically significant molluscs, as our oceans warm due to climate change, is a global priority. South eastern Australia receives warm water in a strengthening East Australia Current and so resident species are vulnerable to elevated temperature and marine heat waves. This study tested whether prior exposure to elevated temperature can enhance resilience of oysters to ocean warming. Two Australian species, the flat oyster, *Ostrea angasi*, and the Sydney rock oyster, *Saccostrea glomerata*, were obtained as adults and “heat shocked” by exposure to a dose of warm water in the laboratory. Oysters were then transferred to elevated seawater temperature conditions where the thermal outfall from power generation was used as a proxy to investigate the impacts of ocean warming. Shell growth, condition index, lipid content and survival of flat oysters and condition of Sydney rock oysters were all significantly reduced by elevated seawater temperature in the field. Flat oysters grew faster than Sydney rock oysters at ambient temperature, but their growth and survival was more sensitive to elevated temperature. “Stress inoculation” by heat shock did little to ameliorate the negative effects of increased temperature, although the survival of heat-shocked flat oysters was greater than non-heat shocked oysters. Further investigations are required to determine if early exposure to heat stress can enhance resilience of oysters to ocean warming.

## Introduction

Climate change, the result of anthropogenic activities such as the burning of fossil fuels and deforestation, has exponentially increased the concentration of carbon dioxide (CO_2_) and other greenhouse gasses in the atmosphere [1]. Since the onset of the industrial revolution, atmospheric partial pressure of CO_2_ (*p*CO_2_) has increased from 280 ppm to 410 ppm causing global warming with direct impacts on the oceans [1,2]. As a result, the world’s oceans have warmed by 0.68°C and for the East Australian coast are predicted to increase by up to 4°C by 2050 and 6°C before 2100 [3,4]. Ocean warming and the increased incidence of heatwaves (abnormal high temperatures over multiple days [5]) negatively impacts diverse species [6]. Between 1925 and 2016 there has been a 54% annual increase in the duration of marine heatwaves worldwide [7]. Climate change is also impacting ocean stratification, currents, salinity, pH, sea level and increasing the frequency of extreme events [1,7,8].

Increasing frequency of thermal stress events will have consequences for fitness and survival of marine species and there is particular concern for habitat engineers such as oysters [6,9]. If molluscs are to persist during this century along the southeast coast of Australia and in similar “hot spots” around the globe, they will need to be resilient to marine heat waves and habitat warming. Previous work has found that organisms can build resilience to environmental stress, through prior exposure to stress. [10,11]. Rather like a vaccination, exposure to mild stress in early life has been observed to increase resilience to stress in a diverse array of organisms such as bacteria, plants, insects, mammals and fish [10, 11, 12, 13, 14].

There were greater survival rates in the tidepool fish *Oligocottus maculosus* after exposure to a +12°C heat stress when exposed to subsequent stressful levels of high salinity and low oxygen concentration [10]. The magnitude of the shock and recovery time played an important role in the stress response later in life [10]. Baltic Sea mussels *Mytilus edulis*, exposed to heat shock (+16°C) and then exposed to cadmium (20 μg L^-1^) produced heat shock proteins faster than mussels not exposed to heat stress [15]. Stress resistance may be enabled by production of protective heat shock proteins (e.g. HSP 70), although this is energetically costly. The mechanisms behind stress inoculation, are complex and likely not limited to production of heat shock proteins. Other processes such as alterations in metabolism and epigenetics are also thought to be involved [16, 17].

While mobile species can migrate changing their distribution as the ocean warms, sessile species are vulnerable because they are unable to move and the dispersive larval stages are often short-lived [18,19]. Studies have suggested that sessile organisms such as oysters, which form the basis of aquaculture across the globe, will be impacted by elevated temperature, because of the energetic cost to physiological performance from climate change stress [20, 21]. Significant mortality has been reported for the north American oyster *Crassostrea virginica* exposed to elevated temperature, due to impacts on energetic reserves [22]. Reduced gametogenesis in *M*. *galloprovincialis* has also been directly connected to warming [23]. In oysters, parental exposure to stress (in this case ocean acidification) increased resilience of their larva, and this trait carried over to adulthood [24,25].

The flat oyster, *Ostrea angasi* and the Sydney rock oyster *Saccostrea glomerata* are native to south eastern Australia [26, 27], where they historically formed extensive reefs and are the basis of a USD $30 million aquaculture industry [28,29]. *Saccostrea glomerata* is an intertidal species that occurs along the east and west coast of Australia with a current upper sea surface temperature (SST) range of 24–26°C [28]. *Ostrea angasi* is distributed in shallow subtidal sheltered waterways along a similar range with a current upper SST temperature range of 22–24°C [27], however, this northern (warm) range is likely curtailed by historic overharvesting and introduced parasites in New South Wales (*Polydora* spp.) [27]. *Ostrea angasi* are mostly found sub-tidally in comparatively stable thermal conditions [30]. The distributions of these two species overlap for >1000 kms of eastern Australian coastline and are both currently the focus of reef restoration efforts in the region [31,32]. Both species are known to be vulnerable to acidification [33,34,35] and warming [33,36]. South-eastern Australia receives warm water from the Coral Sea through the southerly flow of the East Australian Current (EAC) [37,38]. The EAC is strengthening and bringing more warm water to south-eastern Australia making the region an ocean warming “hot spot” with sea surface temperatures increasing 3–4 times the global average along with increased incidence of marine heat waves [7,38,39].

The purpose of this study was to test the hypothesis that early exposure to heat stress or heat shock can be used as a mechanism to build resilience of *O*. *angasi* and *S*. *glomerata* to subsequent long-term exposure to warmed seawater. We used the thermal outfall from a power generating station as a proxy for ocean warming conditions as in previous studies eg. [40] where seawater can be warmed 10–15°C above ambient summer temperatures. Due to their different thermal ranges, distributions and habitats we predicted that *S*. *glomerata* would be more resilient than *O*. *angasi* to elevated temperature. As momentum gains to restore oyster reefs [31], knowledge of oyster responses and how to build resilience is needed to ensure sustainability of restoration efforts and the aquaculture industry.

## Methods

Adult (approximately two years old) *Ostrea angasi* and *Saccostrea glomerata* were obtained from an oyster farm at Merimbula Lake (Merimbula Gourmet Oysters; 36°89’ 85”S, 149°88’ 46”E) where the water temperature at collection was 20°C. Approximately 200 oysters per species were transported to Port Stephens Fisheries Institute (PSFI; 32°44’47”S, 152°03’30”E), in New South Wales, Australia, during the Austral autumn 2018. All oyster movements followed the NSW Fisheries Management (Aquaculture) Regulations. The initial mean shell height was 69.68 ± S.E. 0.34 mm for *O*. *angasi* and 69.86 ± S.E. 0.33 mm for *S*. *glomerata*. After arrival at PSFI the oysters were placed in 40 L tubs with seawater supplied from a 750 L tank at 20°C. Oysters were fed a mixture of microalgae cultured on-site containing 50% *Chaetoceros muelleri* and 50% *Tisochrysis lutea* at a concentration equivalent to 2 x 10^9^ cells oyster^-1^ d^-1^ [41]. The initial mean (± S.E.) condition index for *O*. *angasi* and *S*. *glomerata* were 4.12 ± 0.42 g and 4.30 ± 0.39 g (n = 6), respectively (see below for methods).

### Heat shock

To determine if exposure to heat shock would confer subsequent resilience to long term exposure to elevated temperature, the following heat shock protocol was used. The oysters were divided into two sub-groups; one “control” and a “heat shocked” groups per species into 750 L tanks. Heat shock was administered by exposure to an elevated temperature of 26°C for 18 hours and then 28°C for 6 more hours by slowly ramping up the temperature using aquarium heaters (Titan G2 1500 W). This was an initial +6°C (from 20°C to 26°C) and a further increase of +2°C (from 26°C to 28°C). The 28°C maximum represents a 3.5°C increase on mean (last 12 years) summer water temperatures in Port Stephens, and 1°C increase on the maximum summer daily water temperatures on record [42]. There was no mortality following heat shock treatment. Following the 24 hours at elevated temperature, the seawater was returned to ambient (20°C) over a 12-hour period. Oysters were submerged in ambient water overnight in the laboratory. On the following day, all oysters were placed in baskets and left submerged at ambient conditions, in the adjacent estuary of PSFI (Tilligerry creek, Port Stephens) which remained at 20°C for one week. After this period, they were removed and shell height was measured with a digital calliper.

**Fig 1:**
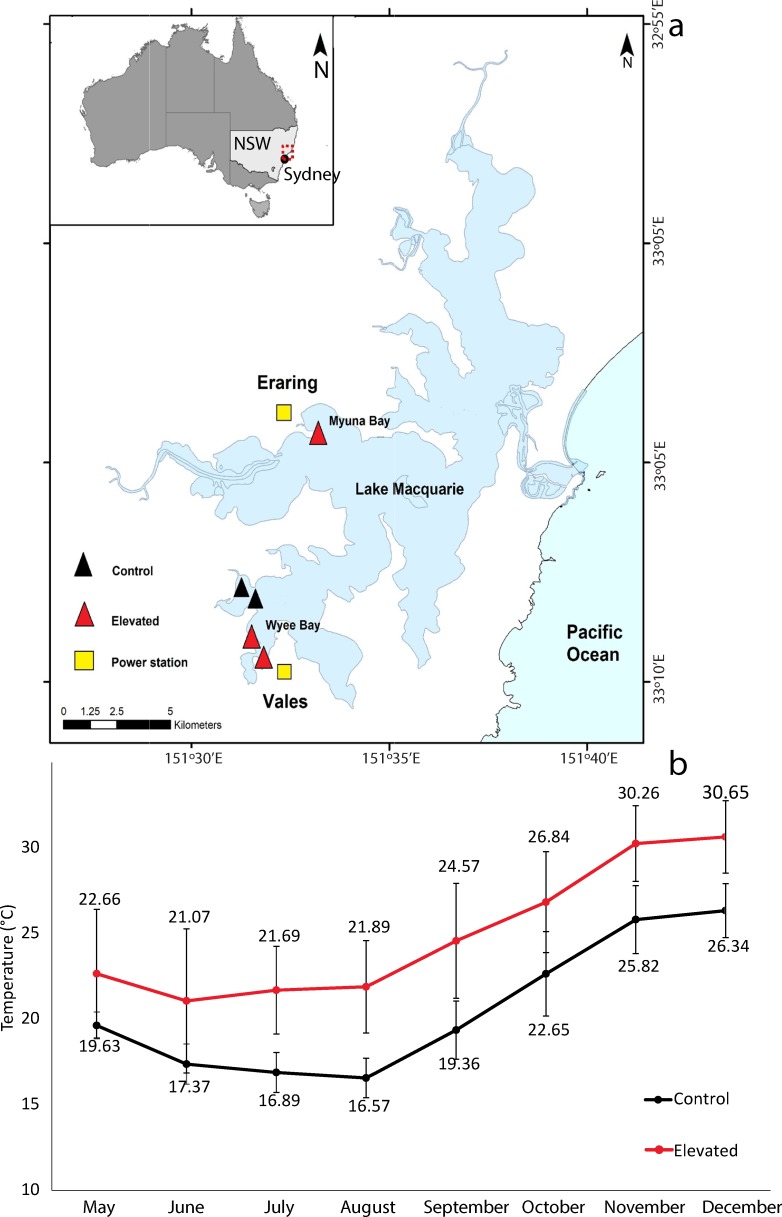
Map of study location and temperatures over study period. **a)** Map of Lake Macquarie, New South Wales (NSW), with map a of Australia (top left) showing the field locations where the baskets were deployed and then retrieved following approximately seven months. Yellow squares represent the warm seawater outfall of two power stations (Eraring and Vales power stations). Black triangles are the ambient (control) locations and the red triangles are the elevated locations (total 5 baskets). **b)** Mean monthly temperatures ± S.D. at control (ambient) and elevated temperature locations in Lake Macquarie, NSW from May to December 2018 (approximately seven months). Temperature data was measured every 30 minutes at 1.10m depth.

A total of 40 oysters were randomly placed into each of six baskets (SEAPA Co. Edwardstown South Australia, 600 x 250 x100 mm) divided into four compartments with 10 “control” *O*.*angasi* and 10 “control” *S*. *glomerata* which were exposed to ~20°C at all times, 10 “heat-shocked” *O*. *angasi* and 10 “heat shocked” *S*. *glomerata*, which were exposed to elevated temperature for 24 hours. 60 heat shocked and 60 non-heat shocked oysters of each species were spread across six baskets. The baskets were transported and deployed into Lake Macquarie (33°.07’94”, 151°.54’85”, Fig 1A).

### Field location

To determine the response of oysters in the real world of elevated seawater temperature, we used warmed seawater released into a saline coastal lake by two power stations at Lake Macquarie, NSW. Lake Macquarie is a large coastal body of water in the centre of east Australian warming “hot spot”. Lake Macquarie is connected to the ocean and has daily tidal exchange. There is little freshwater input from the surrounding catchment [43]. Two coal fired power stations are located 23 kilometres apart on the shore of Lake Macquarie. Eraring power station is located in Myuna Bay (33° 4'2.92"S, 151°33'19.13"E) and Vales Point power station is located in Wyee Bay (33° 9'30.65"S, 151°31'48.37"E). Both stations use seawater from Lake Macquarie for cooling. The seawater is circulated for cooling and then released back into the estuary with no other treatment at a maximum of 37.5°C as per licence requirements (NSW Environment Protection Licences 761; 1429). One location was selected near each power station outfall of the Eraring and Vales Point power stations in Lake Macquarie during May 2018 (autumn). A control location was also selected that represented the ambient mean temperature within Lake Macquarie which was not warmed by a power station. At each location, two baskets were deployed within 20m of each other. Each individual basket was attached to a 10 Kg concrete brick and contained a total of 40 oysters from both species and treatments (control/non heat shock and heat shock) and were deployed at a depth of 1.10 m by boat. A scientific collection permit was obtained from NSW Department of Planning, Investment and Environment (Permit No: F97/109-7.1), which allowed for *S*. *glomerata* and *O*. *angasi* to be deployed in Lake Macquarie.

Temperature data were collected every 30 minutes by Hobo loggers (HOBO MX Pendant Temperature, Onset) which were within the baskets amongst the oysters. Study locations were visited five times over seven months (late autumn to early summer) to download temperature data and renew the loggers. Oysters were deployed in Lake Macquarie for approximately seven months. At the end of the deployment (7 months) five baskets were retrieved; two from the ambient (control) location and three from elevated locations; two from Wyee Bay near Vales power station and one from Rocky Point near Earing power station (120 oysters from elevated temperature and 80 oysters from ambient temperature). One basket was lost at Rocky Point. Once retrieved, oysters were transferred by boat in aerated seawater (collected from the site of retrieval for those oysters) to a temporary processing station (33° 5'8.94"S, 151°30'13.01"E) on the shore of Lake Macquarie. Here, shell growth, condition index, standard metabolic rate and survival of oysters was measured. Total lipid and profile were measured in the laboratory.

### Shell growth and condition index

To determine if exposure to heat shock confers subsequent resilience to long term exposure to elevated temperature on growth and condition index measurements of oysters were done at the end of seven months of exposure in the field experiment.

There was no difference between shell height of oysters randomly allocated into heat shock and control (non- heat shock) treatments (One-way ANOVA comparing heat shock vs non-heat shock for each species, n = 60; *O*. *angasi* = p > 0.05; *S*. *glomerata* = p > 0.05) at day zero. Final shell growth was then calculated as the difference between the final size of each individual oyster at seven months from an overall initial mean size of oysters per basket (n = 10). The difference in shell growth was calculated by the formula:

$$SG=\frac{SH1-MSH0}{t}$$

Where shell growth (SG) is the difference between final individual shell height (SH_1_) in millimetres and the mean initial shell height (MSH_0_) divided by time (t) in days.

The condition index of oysters was measured at the end of the experiment. Oysters were shucked, and body tissue and shell of individuals were dried in oven at 60°C for two days, to determine the dry weight (grams). The condition index (Ci) of oysters was then calculated by the formula [44,45]:

$$Ci=\frac{Dry\;body\;weight\;(g)}{Dry\;shell\;weight\;(g)}x100$$

### Standard Metabolic Rate (SMR)

To determine if exposure to heat shock would confer subsequent resilience to long term exposure to elevated temperature on standard metabolic rate (SMR), the SMR of 9–11 oysters of each species, treatment and basket (total 56 oysters; heat shock and control/non-heat shock) were measured at the end of the experiment using the methods of Parker et al. [33]. Measurements were done adjacent to the locations of collection to minimise stress of transport and to use seawater from Lake Macquarie.

To calculate SMR, oxygen consumption was measured by a closed respirometry system (OXY-10 PreSens, AS1 Ltd, Regensburg, Germany). Seawater was collected from Lake Macquarie and filtered through 0.47 μm glass filter paper before being used to fill respirometry chambers. Respirometers were built to accommodate the maximum oyster size (745ml and 830 ml). Each respirometer was connected to a fibre optic probe for measurement of dissolved oxygen in seawater. The probe was previously calibrated using two O_2_ concentration points (0% and 100% oxygen saturation of seawater) following the methods of Parker et al. [33]. Oysters were gently cleaned of any fouling organisms before placed in filtered seawater (adjusted to the corresponding treatment levels). The time that individuals took to lower the oxygen concentration by 20% (~1.2 O_2_ mg L^-1^) was recorded. Following the procedure of Parker et al. [33], only the time that the oyster is open and actively respiring (determined by observed decreasing oxygen) is used to calculate SMR. This is done to guard against the oyster remaining closed from handling stress. After each trial, each container was rinsed clean with filtered seawater (0.47 μm) and wiped clean with paper towel. After measurement the oysters were removed from the container and shucked to separate body tissues and shell. The tissue was then dried in an oven at 60°C for three days to measure their constant dry body tissue and shell weight in grams (±0.0001g, Analytical Balance Sartorius Research). Standard metabolic rates (SMR) were calculated by the formula:

$$SMR=\frac{Vr(L)\;x\;\Delta CwO_{2}\;(mgO_{2}L^{-1})}{\Delta t\;(h)\;x\;bw(g)}$$

where SMR is the oxygen consumption normalized to 1 g of dry tissue mass (mg O_2_ g^-1^ dry tissue mass h-1, *V*_*r*_ is the volume of the respirometry chamber minus the volume of the oyster (L), *ΔC*_*w*_*O*_*2*_ is the change in water oxygen concentration measured (mg O_2_L^-1^), *Δt* is measuring time (h) and *b*_*w*_ is the dry tissue mass (g) of the oyster.

### Total lipid and lipid profile

To determine if exposure to heat shock and elevated temperature influences energy allocation, total lipid and lipid profiles were analysed. Body tissues of the oysters were placed in centrifuge tubes and frozen for analysis of total lipid content and lipid classes. The tissues were kept at -22°C for transport and then stored at -80°C until analysis. The tissues were then freeze dried (Alpha 1–4 LSCbasic, Martin Christ, Germany) and weighed in a microbalance (±0.0001g; Sartorius CPA225D). Lipids were extracted overnight using a modified Bligh & Dyer [46] one-phase methanol-chloroform-water extraction (2:1:0.8 v/v/v). The phases were separated by the addition of chloroform-water (final solvent ratio, 1:1:0.9 v/v/v methanol-chloroform-water). The total solvent extract (TSE) was concentrated using rotary evaporation at 40°C.

An aliquot of the TSE was analysed using an Iatroscan MK VI TH10 thin- layer chromatography-flame ionization detector (TLC-FID) analyser (Tokyo, Japan) to quantify individual lipid classes [47,48]. Samples were applied in duplicate to silica gel SIII chromarods (5μm particle size) using 1 μl micropipettes. Chromorods were developed in a glass tank lined with pre-extracted filter paper. The primary solvent system used for the lipid separation was hexane-diethyl ether-formic acid (60:15:1.5), a mobile phase resolving non-polar compounds such as steryl ester (SE), triacylglycerol (TAG), free fatty acids (FFA), monoacylglycerol (MAG) and diacylglycerol (DAG). After development, the chromorods were oven dried and analysed immediately to minimize absorption of atmospheric contaminants. The FID was calibrated for each compound class (phosphatidylcholine (PL), cholesterol (Chol), cholesteryl palmitate (SE), palmitic acid (FFA), monopalmitin (MAG), dipalmitin (DAG), tripalmitin (TAG)). Peaks were quantified on an IBM compatible computer using DAPA Scientific software (Kalamunda, Western Australia, Australia). TLC-FID results are generally reproducible with a coefficient of variance of up to 3.46% of individual class abundances [49].

### Survival

Oyster survival was determined after seven months deployment by emptying baskets one section at a time (to avoid mixing) and counting the total number of live oysters.

### Data analysis

Statistical analyses were done using PRIMER v6+ software using either a three or two factor nested PERMANOVA (PRIMER v6+). This analysis was selected because it is robust to unbalanced designs [50].

For shell growth, condition index, SMR, and total lipids, data were analysed using a three factor PERMANOVA with “heat shock” as fixed factor with two levels (heat shock or control), “temperature” as fixed factor with two levels (ambient and elevated), and “basket” as random factor with two levels (basket 1 and basket 2) nested in temperature and heat shock. The analysis used 9999 permutations and alpha was set at *P* < 0.05 to be considered as statistically different. The percentage survival at seven months was analysed using a two factor PERMANOVA with heat shock as fixed factor with two levels (heat shock or control) and temperature as fixed factor with two levels (ambient and elevated).

The composition of lipid profiles were fourth root transformed to limit the influence of large numbers [50] and analysed using a four factor multivariate PERMANOVA using the same model as above; with heat shock as fixed factor with two levels (Heat shock or control), temperature as fixed factor with two levels (control and elevated), and basket as random factor with two levels (basket 1 and basket 2) nested in temperature and heat shock. The analysis used 9999 permutations and alpha was set at *P* < 0.05 to be considered as statistically different. Tabulated results for all statistical tests can be found in S1–S3 Tables.

## Results

### Temperature

The average temperature over seven months at the ambient location was 20.06°C ± 3.85 (mean ± S.D.) and the average temperature at the elevated locations (Wyee Bay, Rocky Point) was 24.56°C ± 4.59 (Fig 1B). The highest daily temperature experienced by oysters deployed at elevated temperature locations was 32.81 ± 0.39°C in spring (November) and lowest daily average for the ambient location during the experiment was 14.92°C ± 0.65 (mean ± S.D) in winter (August).

### Shell growth and condition index

**Fig 2:**
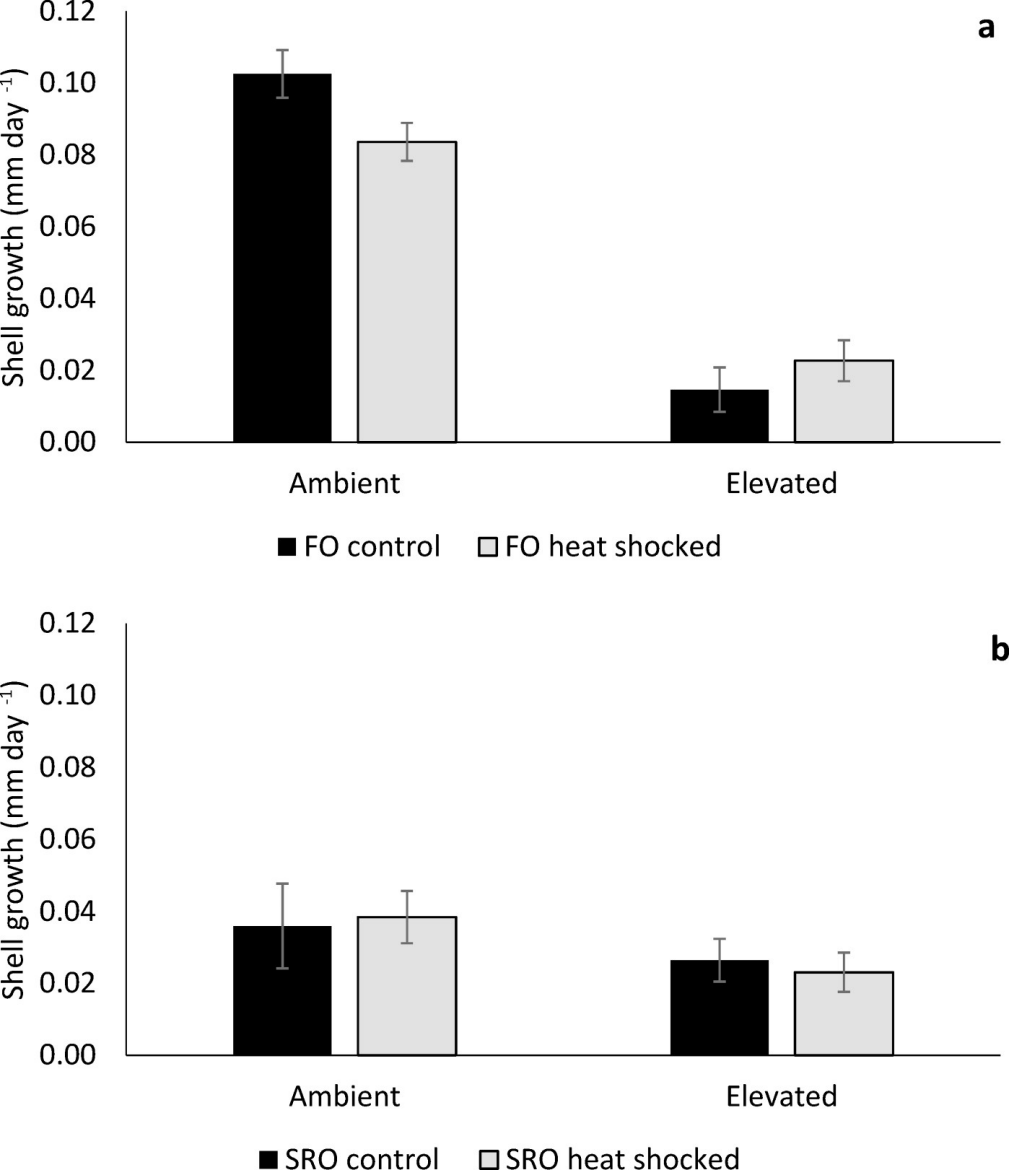
Shell growth of flat and Sydney rock oysters. Mean difference in shell growth (± S.E.) for **a.** flat oysters, *Ostrea angasi* (FO control and FO heat shocked) and **b.** Sydney rock oysters, *Saccostrea glomerata* (SRO control and SRO heat shocked), exposed for seven months at ambient and elevated temperature locations at Lake Macquarie.

**Fig 3:**
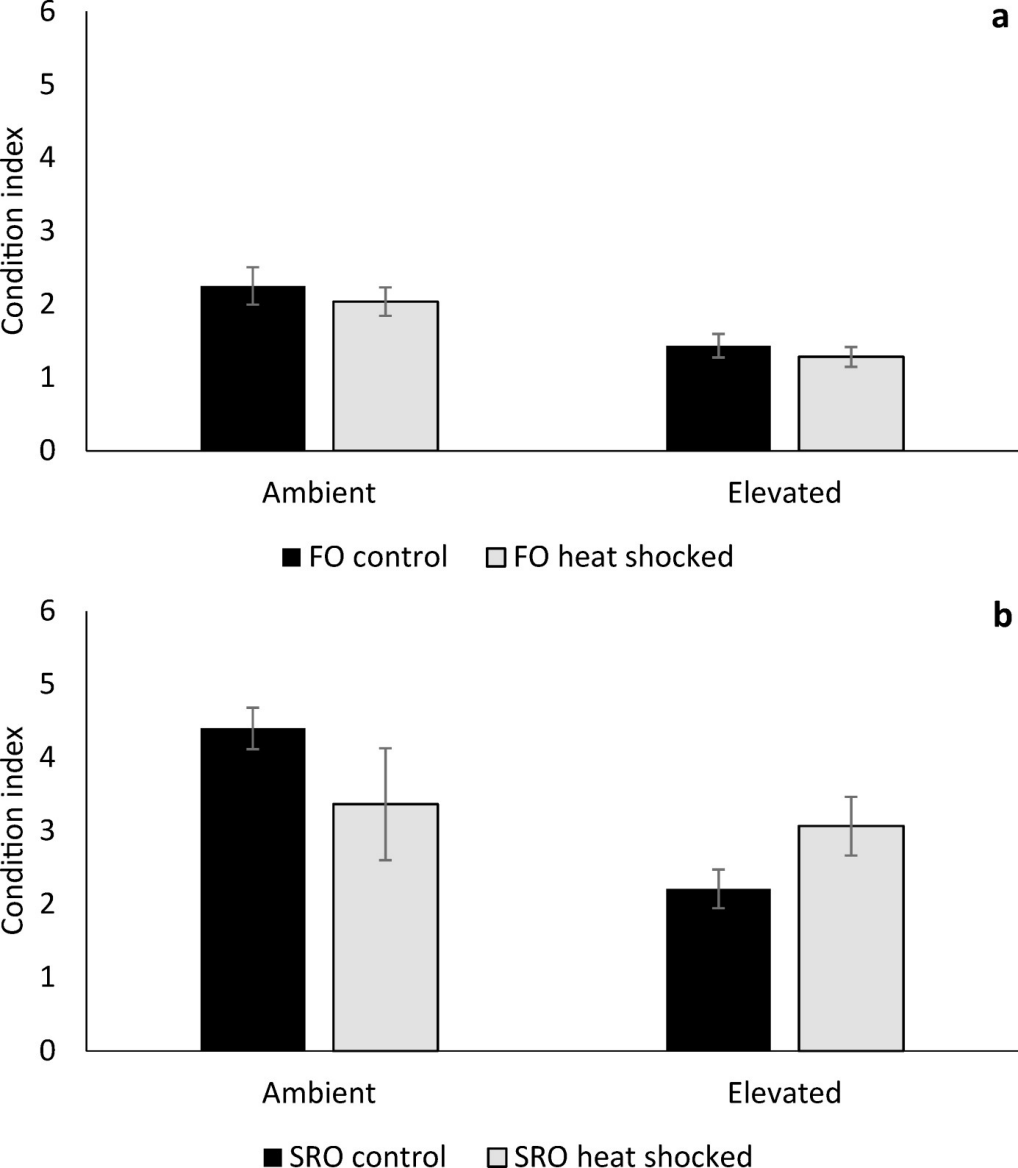
Condition index of flat and Sydney rock oysters. Mean condition index (± S.E.) of **a.** flat oysters, *Ostrea angasi* (FO control and FO heat shocked) and **b.** Sydney rock oysters, *Saccostrea glomerata* (SRO control and SRO heat shocked) exposed for seven months at ambient and elevated locations at Lake Macquarie.

Shell growth of *O*. *angasi* was almost ten-fold greater at ambient temperature compared to elevated temperature (Fig 2A). At ambient temperatures, mean shell growth (mm day^-1^) of non-heat shocked flat oysters was 0.10 ± 0.01 mm day^-1^ (mean ± S.E), which was significantly greater than heat shocked oysters, which grew 0.08 ± 0.01 mm day^-1^ (mean ± S.E). (Fig 2A, PERMANOVA; Heat shock x Temperature: F_4,69_ = 25.84, *P* < 0.001). At elevated temperatures, mean shell growth (mm day^-1^) of non-heat shocked flat oysters was 0.01 ± 0.001 mm day^-1^ (mean ± S.E) compared to heat shocked flat oysters which grew 0.02 ± 0.01 mm day^-1^ (Fig 2A). Shell growth of *S*. *glomerata* was not affected by temperature or heat shock (Fig 2B, PERMANOVA; *P* > 0.05). *Ostrea angasi* grew an order of magnitude greater than *S*. *glomerata* under ambient conditions, however, under elevated temperature there was little growth of either species < 0.04 mm day^-1^ (mean ± S.E) (Fig 2A and 2B). The condition index of both *O*. *angasi* (PERMANOVA; Temperature: F_1, 28_ = 7.87, *P* = 0.04) and *S*. *glomerata* (PERMANOVA; Temperature: F_1, 19_ = 8.29, *P* = 0.02) was significantly lower at elevated temperature (Fig 3A and 3B) with no effect of heat shock treatment.

### Standard Metabolic Rate (SMR)

**Fig 4:**
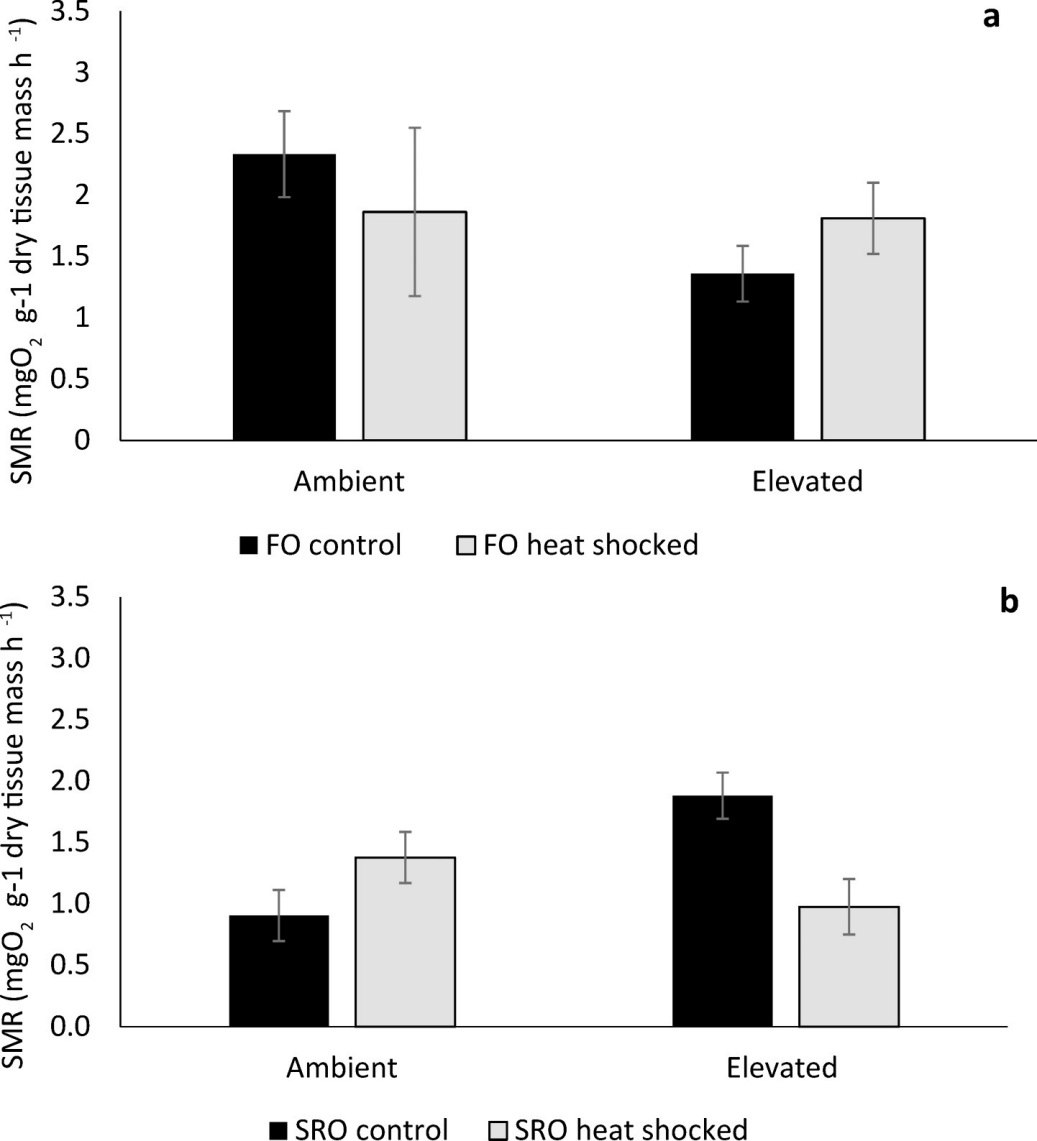
Standard metabolic rate (SMR) of flat and Sydney rock oysters. Mean standard metabolic rate (SMR) (± S.E.) of **a** flat oysters, *Ostrea angasi* (FO control and FO heat shocked) and **b.** Sydney rock oysters, *Saccostrea glomerata* (SRO control and SRO heat shocked) exposed for seven months at ambient and elevated temperature locations at Lake Macquarie.

Standard Metabolic Rate of non-heat shocked *O*. *angasi* was greater at ambient temperature, compared to elevated temperature, but this was not significant (Fig 4A; PERMANOVA; *P >* 0.05). SMR of, non-heat shocked *S*. *glomerata* was greater at elevated, compared to ambient temperature, but this was also not significant (Fig 4B; PERMANOVA; *P >* 0.05).

### Total lipids and lipid profiles

**Fig 5:**
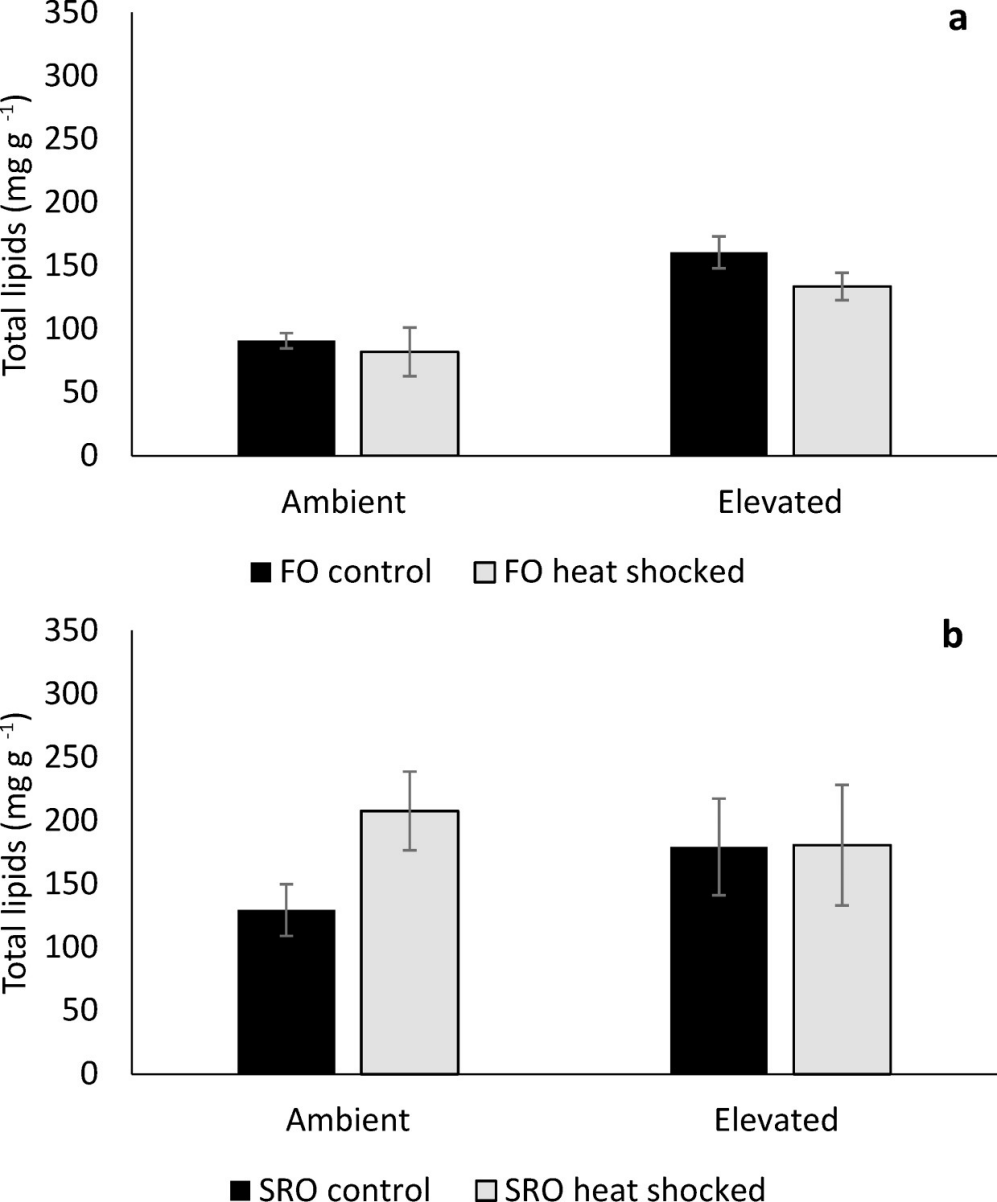
Total lipids of flat and Sydney rock oysters. Mean total lipids (± S.E.) for **a.** flat oysters, *Ostrea angasi* (FO control and FO heat shocked) and **b.** Sydney rock oysters, *Saccostrea glomerata* (SRO control and SRO heat shocked) exposed for seven months at ambient and elevated temperature locations (n = 5; except for HS oysters from Rocky Point–FO HS [n = 3], SRO HS [n = 2]) at Lake Macquarie.

**Fig 6:**
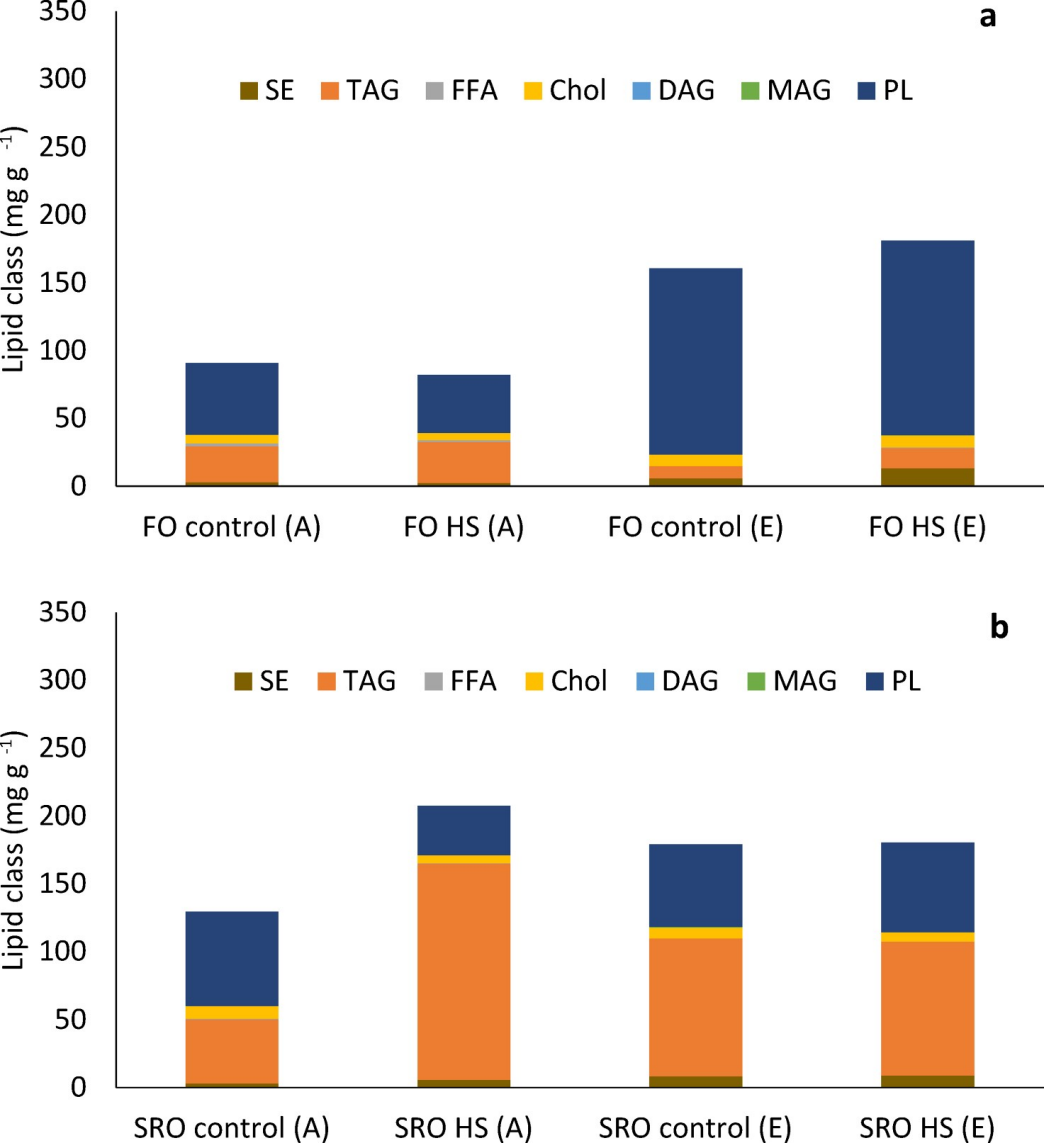
Lipid profiles of flat and Sydney rock oysters. Lipid profile of **a.** flat oysters, *Ostrea angasi* (FO control FO heat shocked) after seven months exposure at ambient and elevated temperature locations and **b.** Lipid profile of Sydney rock oysters *Saccostrea glomerata* (SRO control and SRO heat shocked) exposed for seven months at ambient and elevated temperature. Lipid classes abbreviations are: SE–steryl ester; TAG -Triacyglyceride; FFA–Free Fatty Acids; Chol–Cholesterol; DAG–Diacylglyceride; MAG–Monoglyceride and PL–Phospholipids.

The total lipid content of *O*. *angasi* was greater at elevated compared to ambient temperature (Fig 5A, PERMANOVA; Temperature: F_1, 14_ = 7.55, *P* = 0.03). There were no effects of heat shock or temperature on total lipid content in *S*. *glomerata* (Fig 5B, *P >* 0.05). The lipid profile of *O*. *angasi*, was mostly driven by a significantly greater amount of phospholipids in oysters at elevated temperature (Fig 6A, PERMANOVA; Temperature: F_1, 15_ = 13.21, *P*< 0.001) and lower amounts of triacylglyceride (TAG) (Fig 6A) irrespective of heat shock. The changes in lipid profile of *S*. *glomerata* was similar across ambient and elevated temperatures, but there were significantly greater phospholipids at elevated temperature (Fig 6B, PERMANOVA; Temperature: F_1, 17_ = 7.31, *P* = 0.04).

### Survival

**Fig 7:**
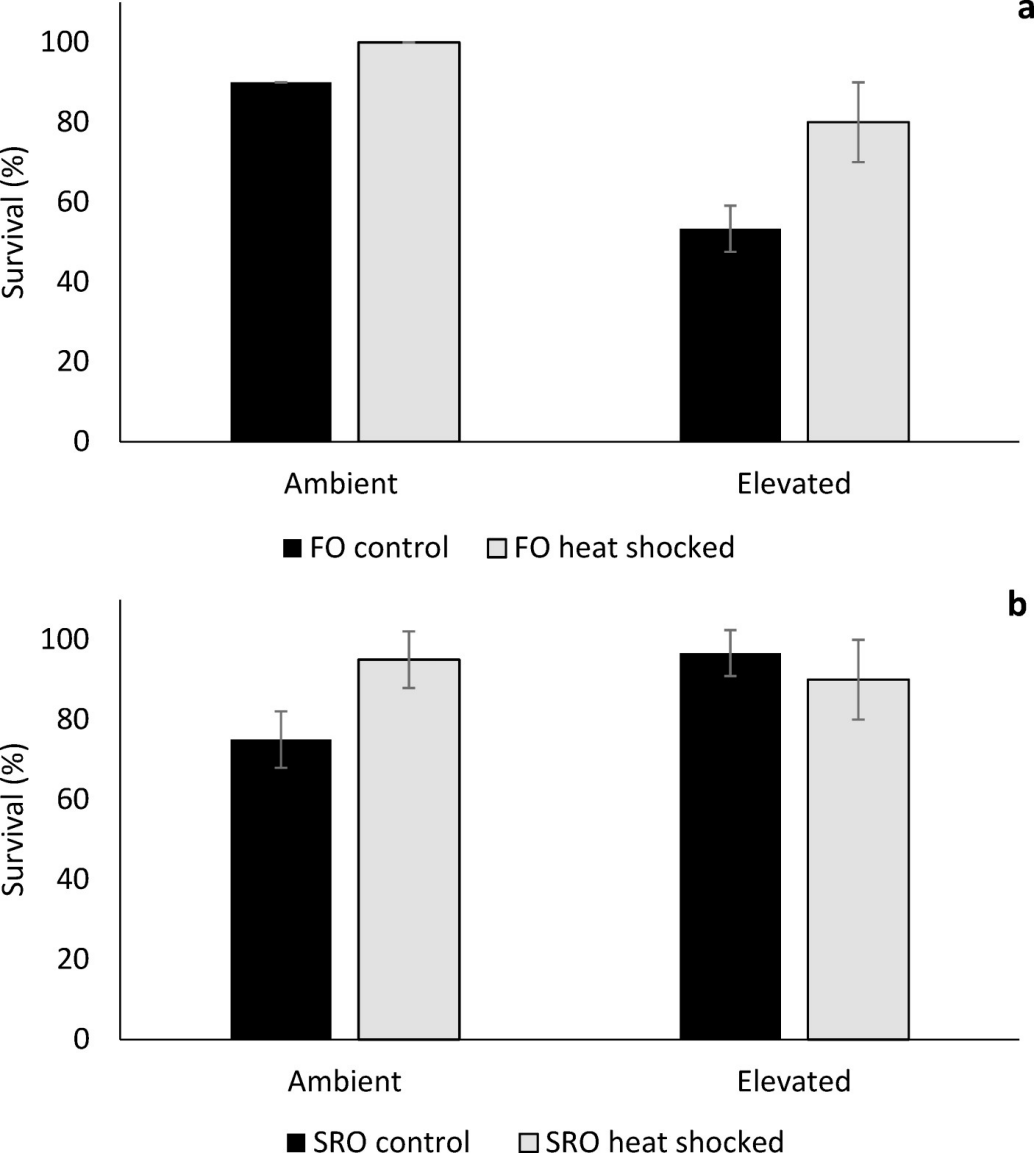
Survival of flat and Sydney rock oysters. Mean survival (±S.D.) of **a** flat oysters, *Ostrea angasi* (FO control and FO heat shocked) and **b.** Sydney rock oysters, *Saccostrea glomerata*, (SRO control and SRO heat shocked), exposed for seven months at ambient and elevated temperature locations at Lake Macquarie.

Survival of heat-shocked *O*. *angasi* was significantly greater than non-heat shocked oysters at ambient and elevated temperature (Fig 7A; PERMANOVA; Heat shock x Temperature: F_1, 6_ = 16.61, *P =* 0.04). At the ambient and elevated temperature locations, survival of *O*. *angasi* was greatest for the heat shocked oysters (Ambient, control oysters = 90% and heat shocked oysters = 100%; Elevated, control = 53% and heat shocked = 80%). Survival of *S*. *glomerata* was significantly lower for non-heat shocked oysters at ambient temperature compared to heat shocked oysters (Fig 7B, PERMANOVA; Heat shock x Temperature: F_1, 6_ = 16.61, *P =* 0.04).

## Discussion

Exposure to long term warming in the field had negative impacts on shell growth, condition index, and survival of *O*. *angasi* and *S*. *glomerata*. Shell growth, condition index, lipid content and profile and survival, but not SMR of *O*. *angasi* were impacted by elevated temperature. Only survival and condition index of *S*. *glomerata* was negatively impacted by elevated temperature. Flat oysters grew faster than Sydney rock oysters at ambient temperature but were more sensitive to elevated temperature. Prior exposure to heat shock did little to ameliorate the negative effects of elevated temperature. Heat shocked flat oysters, however had greater survival at elevated temperature. There was also a trend for shell growth of heat shocked flat oysters to be greater than non-heat shocked oysters at elevated temperature. SMR was not significantly impacted by elevated temperature, although once again there was a trend for SMR of flat oysters to decrease with increased temperature and for SMR of Sydney rock oysters to increase with increased temperature. The levels of TAG within the lipid profile of *O*. *angasi* was also reduced by elevated temperature, while the lipid profile for *S*. *glomerata* was not affected.

As oysters are ectothermic organisms, changes in external temperature away from their optimum causes physiological processes to become less efficient and homeostasis begins to require more energy [51]. The effects of elevated temperature on *O*. *angasi* and to some extent, *S*. *glomerata* are similar to those observed for other bivalve species. For example, Hiebenthal et al. [52] found lower growth and condition for *Arctica islandica* at elevated temperature (16°C) compared to the control (7.5°C) and an intermediate treatment (10°C). Condition index and survivorship of *M*. *edulis* was reduced under elevated temperature (25°C) compared with control [52]. Effects on these physiological processes, were attributed to thermal sensitivity of *A*. *islandica* to temperatures outside its distribution and to accumulation of lipofuscin, a disease related pigment [52]. Juvenile shell growth (mg d^-1^) of the clam *Mercenaria mercenaria* and *Argopecten irradians*, was significantly reduced by a 4°C increase in temperature (28°C) [53].

Elevations in temperature increase the SMR of marine ectotherms until a point known as the “Arrhenius Breakpoint Temperature” (ABT). When ABT is reached, SMR rapidly declines indicating that the organism can no longer meet their energetic requirements at that temperature [33]. The reduced growth of *O*. *angasi* at elevated compared to ambient temperature correlated with a trend for lower SMR, indicating that *O*. *angasi* may have experienced temperatures beyond their ABT. In this study, we found the temperatures reached at the thermal outfalls in Lake Macquarie (mean = 24.56 ±4.59, daily max = 32.81 ± 0.39°C) had no effect on the SMR of *S*. *glomerata*. Parker et al., [33] found that increased seawater temperature increased the SMR of *S*. *glomerata*. SMR of *S*. *glomerata* increased with increased temperature up to 33°C (the upper temperature treatment in that study) indicating an ABT for *S*. *glomerata* of above 33°C. Increased SMR can impact energy budget and may indicate a thermal response with extra costs needed to cover basal metabolism [33,54]. For oysters, thermal stress can also alter cardiac function, protein synthesis [54] and gametogenesis [23].

Oysters have the capacity to store surplus energy ingested from food in the form of lipids which can assist in the persistence during stressful conditions. While the lipid profile of *S*. *glomerata* was not impacted by elevated temperature, there were significant impacts of elevated temperature on total lipids and lipid profile, especially triacylglycerides (TAG) of *O*. *angasi*. Lower lipid content may cause lower condition index of *O*. *angasi* at elevated temperatures. TAG are the primary source of stored lipid energy for bivalves [54, 55], indicating that *O*. *angasi* had begun to use stored lipid reserves. Under stressful conditions bivalves have lower lipid reserves. For example, exposure to elevated *p*CO_2_ decreased the lipid index of larvae of *A*. *irradians*, *M*. *mercenaria and C*. *virginica* which further declined when combined with warming [53, 56]. Lipid levels in the eggs of *S*. *glomerata* also decreased when exposed to the dual stress of elevated *p*CO_2_ and copper [57]. Habitat warming may decrease the lipid content, condition index and value of oysters further reducing the profitability of aquaculture [19]. Research on how lipids are used by oysters in response to stress could provide insights into the ramifications of living in warmer oceans.

### Stress inoculation, and resilience

This study tested the hypothesis that prior-exposure of oysters to heat shock stress will build resilience to later exposure to elevated temperature. Stress inoculation leading to stress resilience has been observed in diverse phyla from bacteria to mammals e.g. [10,11]. Heat shock may help to build resilience, but at the same time have costs. For example, heat shocked *O*. *angasi* had significantly greater rates of survival at elevated temperatures, but heat shocked oysters had less growth at ambient temperature. Energy may have been used to produce heat shock proteins or other protective measures, thereby reducing energetic reserves for growth and other important physiological processes [58].

Heat shocked *O*. *angasi* had greater rates of survival at elevated temperature, and a similar trend was observed for *S*. *glomerata* which had greater than 90% survival at elevated temperature. When organisms experience stressful temperatures, they undergo a thermal response, which is energy dependent [58]. This thermal response includes producing chaperone proteins, such as energetically expensive HSPs [58,59]. Species with lower thermal tolerance might be induced to produce HSPs in response to elevated temperatures at lower levels of warming than more tolerant species, which can endure longer periods of heat stress (e.g. *M*. *trossulus* and *M*. *galloprovincialis;* [59]). Heat shock proteins have important functions when an organism is exposed to elevated temperature, including degradation of denatured proteins and prevention of misfolding, having a key function on cellular protection [60]. These responses (e.g. expression of heat shock proteins, antioxidants, increased respiration rates) all incur an energetic cost which can cause an imbalance in the energetic partitioning of individuals [33,58,61,62].

In this study, the heat shock was administered to adult oysters for a relatively short period of 24 hours. A longer time period may have allowed oysters to produce more heat shock proteins (HSP) or for epigenetic shifts to occur [58, 63]. Furthermore, positive cross-generation effects may have occurred if the heat shock had been administered to the parents of the oysters [64]. Previous research has shown that the resilience of *S*. *glomerata* can be built to environmental stressors such as ocean acidification with transgenerational exposure [24, 25]. This, however, can be limited. Parker et al., [65] found that adaptations were maladaptive under other stressors, including warming [65].

Overall, *S*. *glomerata* was found to be generally more tolerant of habitat warming than *O*. *angasi*. *S*. *glomerata* had no change in shell growth although they were in poorer condition at elevated temperature of 28–30°C. These findings are supported by previous work by Parker et al., [33] that showed 33°C was not beyond their ABT. As an intertidal species that experiences a highly dynamic thermal environment, *S*. *glomerata* could be expected to be more thermally tolerant as has been shown for other intertidal organisms [63,66]. In addition, *S*. *glomerata* is distributed further north along the east coast of Australia than *O*. *angasi* and is likely to have a greater temperature tolerance; *S*. *glomerata* can experience air temperatures in excess of 40°C during emersion at low tide [67]. The lack of effect of elevated temperature on *S*. *glomerata* indicates that the temperatures experienced in this study (heat shock; 28°C, max outfall daily max; 32.81 ± 0.39°C) did not place *S*. *glomerata* beyond their thermal limits in contrast to *O*. *angasi*, which did not cope as well.

*O*. *angasi* had the greatest growth rate at ambient conditions. The shell growth of *O*. *angasi* was over ten-fold greater than Sydney rock oysters after seven months, as expected from growth in aquaculture [68]. While *O*. *angasi* grew well at ambient conditions, growth and survival were impacted by warming. As this species lives in a relatively stable, sub-tidal habitat we expected this species to be more sensitive to warming compared to *S*. *glomerata*.

Globally and across Australia efforts are being made to restore oyster reefs [31,69,70]. Climate change will impact on oyster reef restoration [36]. Projected ocean warming for the region (4°C) as well as contemporary marine heat waves, as seen in the region recently [39] are an important consideration for reef restoration efforts in southeast Australia. Our study has shown that using a thermal outfall as a proxy for ocean warming can be useful in assessing organism responses to projected habitat warming. This approach is similar to natural laboratories using underwater CO_2_ vents which have successfully tested the responses of marine organisms to ocean acidification [71,72]. Our results indicate that habitat warming will be a greater threat to *O*. *angasi* than *S*. *glomerata*. As ocean warming will not act alone, oyster reef restoration is at risk from multiple stressors including ocean acidification, salinity, and other environmental pollutants which will act simultaneously [36]. These co-occurring stressors further threaten native species of oysters and other marine organisms and so mitigation strategies to build oyster resilience will be critical. Our results indicate that early exposure to stress inoculation does not enhance resilience and may not be useful strategy, especially for restoration ventures involving *O*. *angasi*.

## Supporting information

S1 Table **a.** Results of PERMANOVA for the shell growth, condition index and SMR of *Ostrea angasi* exposed for seven months in Lake Macquarie. **b.** Results of PERMANOVA for the shell growth, condition index and SMR of *Saccostrea glomerata* exposed for seven months in Lake Macquarie. P values were created using Monte Carlo tests. Significant values (P<0.05) are bold.
(DOCX)

S2 Table **a.** Results of PERMANOVA for the total lipids, amount of total lipids (mg/g), amount of Triacylglycerides (TAGs; mg/g) and Phospholipids (PLs; mg/g) of *Ostrea angasi* exposed for seven months in Lake Macquarie. **b.** Results of PERMANOVA for the total lipids (mg/g), amount of Triacylglycerides (TAGs; mg/g) and Phospholipids (PLs; mg/g), of *Saccostrea glomerata* exposed for seven months in Lake Macquarie. P values were created using Monte Carlo tests. Significant values (P<0.05) are bold.
(DOCX)

S3 Table **a.** Results of PERMANOVA for the percentage survival of *Ostrea angasi* exposed for seven months in Lake Macquarie. **b.** Results of PERMANOVA for the percentage survival of *Saccostrea glomerata* exposed for seven months in Lake Macquarie. P values were created using Monte Carlo tests. Significant values (P<0.05) are bold.
(DOCX)

S1 Raw data (CSV)
