# Supplementary material for: Can prior exposure to stress enhance resilience to ocean warming in two oyster species?
Source: PLoS One. 2020 Apr 10;15(4):e0228527. doi: 10.1371/journal.pone.0228527 (PMC7147797; doi:10.1371/journal.pone.0228527)
Supplement: S1 Table — a. Results of PERMANOVA for the shell growth, condition index and SMR of Ostrea angasi exposed for seven months in Lake Macquarie. b. Results of PERMANOVA for the shell growth, condition index and SMR of Saccostrea glomerata exposed for seven months in Lake Macquarie. P values were created using Monte Carlo tests. Significant values (P<0.05) are bold. (DOCX) [file pone.0228527.s001.docx]

**S1 Table a.** Results of PERMANOVA for the shell growth, condition index and SMR of *Ostrea angasi* exposed for seven months in Lake Macquarie. P values were created using Monte Carlo tests. Significant values (P<0.05) are bold.

|  | **Shell Growth** | | | | **Condition index** | | | | **SMR** | | | |
| --- | --- | --- | --- | --- | --- | --- | --- | --- | --- | --- | --- | --- |
|  | df | MS | Pseudo-F | P(MC) | df | MS | Pseudo-F | P(MC) | df | MS | Pseudo-F | P(MC) |
| Heat Shock | 1 | 9.97 | 1.55 | 0.27 | 1 | 166.93 | 0.42 | 0.65 | 1 | 241.95 | 0.19 | 0.78 |
| Temperature | 1 | 3960 | 616.92 | **<0.001** | 1 | 3201.8 | 7.87 | **0.04** | 1 | 1748.70 | 1.36 | 0.3 |
| Heat Shock x Temperature | 1 | 165.88 | 25.84 | **<0.001** | 1 | 626.55 | 1.55 | 0.29 | 1 | 701.91 | 0.54 | 0.54 |
| Basket (Heat Shock x Temperature) | 4 | 5.91 | 0.2 | 0.94 | 3 | 402.87 | 1.56 | 0.20 | 4.00 | 1337.00 | 2.46 | 0.06 |
| Residuals | 69 | 29.23 |  |  | 28.00 | 258.09 |  |  | 20.00 | 543.10 |  |  |
| Total | 76 |  |  |  | 34.00 |  |  |  | 27.00 |  |  |  |

**S1 Table b**. Results of PERMANOVA for the shell growth, condition index and SMR of *Saccostrea glomerata* exposed for seven months in Lake Macquarie. P values were created using Monte Carlo tests. Significant values (P<0.05) are bold.

|  | **Shell Growth** | | | | **Condition index** | | | | **SMR** | | | |
| --- | --- | --- | --- | --- | --- | --- | --- | --- | --- | --- | --- | --- |
|  | df | MS | Pseudo-F | P(MC) | df | MS | Pseudo-F | P(MC) | df | MS | Pseudo-F | P(MC) |
| Heat Shock | 1 | 2.60 | 0.04 | 0.85 | 1 | 132.82 | 0.42 | 0.65 | 1 | 102.31 | 0.12 | 0.90 |
| Temperature | 1 | 186.07 | 2.84 | 0.17 | 1 | 2651.80 | 8.29 | **0.02** | 1 | 1181.50 | 1.39 | 0.29 |
| Heat Shock x Temperature | 1 | 0.00 | 0.00 | 0.99 | 1 | 777.29 | 2.43 | 0.16 | 1 | 1571.90 | 1.85 | 0.21 |
| Basket (Heat Shock x Temperature) | 4 | 66.49 | 1.49 | 0.22 | 4 | 319.59 | 0.96 | 0.46 | 4 | 880.30 | 1.38 | 0.24 |
| Residuals | 79 | 44.58 |  |  | 19 | 331.50 |  |  | 18 | 635.75 |  |  |
| Total | 86 |  |  |  | 26 |  |  |  | 25 |  |  |  |
