# Supplementary material for: Can prior exposure to stress enhance resilience to ocean warming in two oyster species?
Source: PLoS One. 2020 Apr 10;15(4):e0228527. doi: 10.1371/journal.pone.0228527 (PMC7147797; doi:10.1371/journal.pone.0228527)
Supplement: S2 Table — a. Results of PERMANOVA for the total lipids, amount of total lipids (mg/g), amount of Triacylglycerides (TAGs; mg/g) and Phospholipids (PLs; mg/g) of Ostrea angasi exposed for seven months in Lake Macquarie. b. Results of PERMANOVA for the total lipids (mg/g), amount of Triacylglycerides (TAGs; mg/g) and Phospholipids (PLs; mg/g), of Saccostrea glomerata exposed for seven months in Lake Macquarie. P values were created using Monte Carlo tests. Significant values (P<0.05) are bold. (DOCX) [file pone.0228527.s002.docx]

**S2 Table a.** Results of PERMANOVA for the total lipids, amount of total lipids (mg/g), amount of Triacylglycerides (TAGs; mg/g) and Phospholipids (PLs; mg/g) of *Ostrea angasi* exposed for seven months in Lake Macquarie. P values were created using Monte Carlo tests. Significant values (P<0.05) are bold.

|  | **Total lipids** | | | | **TAGs** | | | | **PLs** | | | |
| --- | --- | --- | --- | --- | --- | --- | --- | --- | --- | --- | --- | --- |
|  | df | MS | Pseudo-F | P(MC) | df | MS | Pseudo-F | P(MC) | df | MS | Pseudo-F | P(MC) |
| Heat Shock | 1 | 204.36 | 0.58 | 0.55 | 1 | 3.58 | 0.02 | 0.96 | 1 | 780.33 | 1.20 | 0.36 |
| Temperature | 1 | 2652.8 | 7.55 | **0.03** | 1 | 798.48 | 3.65 | 0.13 | 1 | 8619.40 | 13.21 | **<0.001** |
| Heat Shock x Temperature | 1 | 270.63 | 0.77 | 0.48 | 1 | 72.98 | 0.33 | 0.62 | 1 | 347.69 | 0.53 | 0.64 |
| Basket (Heat Shock x Temperature) | 4 | 351.92 | 1.47 | 0.24 | 4 | 219.52 | 1.81 | 0.17 | 4 | 653.35 | 1.2 | 0.32 |
| Residuals | 14 | 239.88 |  |  | 15 | 121.39 |  |  | 15 | 545.05 |  |  |
| Total | 21 |  |  |  | 2 |  |  |  | 22 |  |  |  |

**S2 Table b.** Results of PERMANOVA for the total lipids (mg/g), amount of Triacylglycerides (TAGs; mg/g) and Phospholipids (PLs; mg/g), of *Saccostrea glomerata* exposed for seven months in Lake Macquarie. P values were created using Monte Carlo tests. Significant values (P<0.05) are bold.

|  | **Total lipids** | | | | **TAGs** | | | | **PLs** | | | |
| --- | --- | --- | --- | --- | --- | --- | --- | --- | --- | --- | --- | --- |
|  | df | MS | Pseudo-F | P(MC) | df | MS | Pseudo-F | P(MC) | df | MS | Pseudo-F | P(MC) |
| Heat Shock | 1 | 630.71 | 0.62 | 0.51 | 1 | 355.76 | 0.63 | 0.48 | 1 | 380.53 | 2.15 | 0.20 |
| Temperature | 1 | 585.15 | 0.57 | 0.54 | 1 | 40.98 | 0.07 | 0.86 | 1 | 1291.40 | 7.31 | **0.04** |
| Heat Shock x Temperature | 1 | 824.63 | 0.81 | 0.44 | 1 | 951.68 | 1.70 | 0.26 | 1 | 957.97 | 5.42 | 0.06 |
| Basket (Heat Shock x Temperature) | 4 | 1023.70 | 1.50 | 0.22 | 4 | 564.66 | 1.68 | 0.20 | 4 | 172.28 | 0.37 | 0.84 |
| Residuals | 17 | 682.38 |  |  | 17 | 337.06 |  |  | 17 | 461.41 |  |  |
| Total | 24 |  |  |  | 24 |  |  |  | 24 |  |  |  |
