# Supplementary material for: Can prior exposure to stress enhance resilience to ocean warming in two oyster species?
Source: PLoS One. 2020 Apr 10;15(4):e0228527. doi: 10.1371/journal.pone.0228527 (PMC7147797; doi:10.1371/journal.pone.0228527)
Supplement: S3 Table — a. Results of PERMANOVA for the percentage survival of Ostrea angasi exposed for seven months in Lake Macquarie. b. Results of PERMANOVA for the percentage survival of Saccostrea glomerata exposed for seven months in Lake Macquarie. P values were created using Monte Carlo tests. Significant values (P<0.05) are bold. (DOCX) [file pone.0228527.s003.docx]

**S3 Table a.** Results of PERMANOVA for the percentage survival of *Ostrea angasi* exposed for seven months in Lake Macquarie. P values were created using Monte Carlo tests. Significant values (P<0.05) are bold.

|  |  |  |  |  |
| --- | --- | --- | --- | --- |
|  | df | MS | Pseudo-F | P(MC) |
| Heat Shock | 1 | 359.59 | 16.16 | **0.007** |
| Temperature | 1 | 802.23 | 36.05 | **0.001** |
| Heat Shock x Temperature | 1 | 142.45 | 6.40 | **0.04** |
| Residuals | 6 | 22.25 |  |  |
| Total | 9 |  |  |  |

**S3 Table b.** Results of PERMANOVA for the percentage survival of *Saccostrea glomerata* exposed for seven months in Lake Macquarie. P values were created using Monte Carlo tests. Significant values (P<0.05) are bold.

|  | df | MS | Pseudo-F | P(MC) |
| --- | --- | --- | --- | --- |
| Heat Shock | 1 | 39.189 | 2.019 | 0.2037 |
| Temperature | 1 | 57.668 | 2.971 | 0.1309 |
| Heat Shock x Temperature | 1 | 143.35 | 7.3852 | **0.0353** |
| Residuals | 6 | 19.41 |  |  |
| Total | 9 |  |  |  |
